# The spectrum of nodular lymphocyte predominant Hodgkin lymphoma: a report of the lymphoma workshop of the 20th meeting of the European Association for Haematopathology

**DOI:** 10.1007/s00428-023-03554-1

**Published:** 2023-08-02

**Authors:** Sylvia Hartmann, Stefan Dojcinov, Snjezana Dotlic, Sarah E. Gibson, Eric D. Hsi, Wolfram Klapper, Monika Klimkowska, Socorro Maria Rodriguez Pinilla, Julia Richter, Elena Sabattini, Thomas Tousseyn, Daphne de Jong

**Affiliations:** 1 Dr. Senckenberg Institute of Pathology, Goethe University Frankfurt Am Main, Frankfurt Am Main, Germany; 2 Department of Pathology, Morriston Hospital, Swansea Bay University Health Board, Swansea, UK; 3 Dept of Pathology and Cytology, University Hospital Centre Zagreb, Zagreb, Croatia; 4 Division of Hematopathology, Department of Laboratory Medicine and Pathology, Mayo Clinic, Phoenix, AZ USA; 5 Department of Pathology, Wake Forest University School of Medicine, Winston-Salem, USA; 6 Campus Kiel, Department of Pathology, University Hospital Schleswig-Holstein, Hematopathology Section and Lymph Node Registry, Kiel, Germany; 7 Department of Clinical Pathology and Cancer Diagnostics, Karolinska University Hospital Huddinge, Stockholm, Sweden; 8 Pathology Department, Hospital Universitario Fundación Jiménez Díaz, Madrid, Spain; 9 Haematopathology Unit, IRCCS Azienda Ospedaliero-Universitaria Di Bologna, Bologna, Italy; 10 Department of Imaging and Pathology and Translational Cell and Tissue Research Lab, Leuven, Belgium; 11 Dept of Pathology, AmsterdamUMC, Location VUMC, De Boelelaan 1117, 1081HV Amsterdam, The Netherlands

**Keywords:** Nodular lymphocyte predominant Hodgkin Lymphoma, T-cell/histiocyte-rich large B-cell lymphoma, Immune microenvironment, Diagnostic workshop, Gene expression profiling

## Abstract

Session 4 of the 2021 European Association of Haematopathology/Society for Hematopathology Workshop focused on nodular lymphocyte predominant Hodgkin lymphoma (NLPHL). First, the spectrum of immunophenotypic variations in NLPHL and the defining criteria for classic Hodgkin Lymphoma (CHL) were discussed. The added value of further immunophenotypic characterization of both tumor cells and microenvironment to support the differential diagnosis was presented. Next, unusual cases with combined growth patterns and evolution of morphological features over time were presented to explore the clinicopathological impact of presumed high-risk patterns. Based on a large collection of cases, the defining morphological, immunophenotypical, and gene expression features of T-cell/histiocyte-rich large B-cell lymphoma (THRLBCL) and THRLBCL-like NLPHL (pattern E) were reviewed to explore this challenging differential diagnosis and critically evaluate whether aggressive behavior and transformation of NLPHL can be predicted in practice.

**Supplementary information:**

The online version contains supplementary material available at 10.1007/s00428-023-03554-1.

## Introduction

Since the initial descriptions in the early 1980s [1, 2], the morphological spectrum of nodular lymphocyte predominant Hodgkin lymphoma (NLPHL) has expanded significantly and a spectrum of largely architecturally and immunophenotypically defined variations have been described. Specifically, aberrant expression of markers such as CD30 and CD15, an incomplete, deficient B-cell phenotype and EBV-association may result in a challenging differential diagnosis with classic Hodgkin lymphoma (CHL), especially lymphocyte-rich classic Hodgkin lymphoma (LRCHL). Moreover, similarities between both the LP and Hodgkin/Reed-Sternberg (HRS) cells and the composition of the non-malignant immune microenvironment might even suggest a common biology underlying NLPHL and LRCHL. Since standard treatments of NLPHL and CHL are essentially different, accurate distinction between the two diseases is considered essential for effective clinical management and more objective diagnostic criteria to support clinicopathological decision making are needed.

The six growth patterns (“A–F”) described by Fan and co-workers [3] have been incorporated in the WHO classification since 2008, aiding in the recognition and the correct diagnosis of NLPHL particularly in cases with atypical presentations. Generally, patients with variant growth patterns “C–F” significantly more often present with stage IV disease and relapse within the first 5 years after initial diagnosis [3, 4]. However, the clinical impact of the individual growth patterns such as the prominent extra-nodular LP cell pattern (“C”), T-cell rich nodular pattern (“D”), diffuse T-cell/histiocyte-rich large B-cell lymphoma (THRLBCL)-like pattern (“E”), and diffuse moth eaten B-cell rich pattern (“F”) is still largely unknown. Thus far, published studies investigated all variant patterns combined as a single group, precluding definite conclusions on the prognostic impact of individual growth patterns. Decisions on meaningful prognostic subgrouping of variant patterns and associated guidelines for treatment should therefore be considered to be premature [5]. To resolve such clinicopathological questions in a meaningful manner, sufficiently large numbers of cases with rare variant patterns need to be collected and studied, requiring a major (international) effort.

The lymphoma workshop of the 20th meeting of the European Association for Haematopathology (EAHP) held in a virtual format in April 2021 took the opportunity to collect a large case base covering the spectrum of NLPHL and dedicated one whole session to this subject. A total of 63 cases with uncommon morphological and/or immunophenotypical features provided the opportunity to explore the histological spectrum of NLPHL and its differential diagnoses. Twelve cases specifically illustrated involvement of Epstein-Barr virus (EBV), and a possible etiological association was discussed. Various cases illustrated difficulties in demarcating the borderline with CHL based on aberrant tumor cell immunophenotype and composition of the background reactive microenvironment. The second part of the session included 40 cases with uncommon and combined Fan patterns. Since the majority of the cases were well-documented including information on treatment and outcome, this unique series not only illustrated the difficulty separating NLPHL pattern “E” from THRLBCL using currently available criteria, but also provided an opportunity for further study of these cases using newly described immunohistochemical markers and gene expression profiling. Hereby, hypothesis-generating information on prediction of aggressive clinical behavior could be proposed by the workshop panel. It should be noted that the preparations for the updates of the revised 4th edition of the World Health Organization (WHO) Classification started only after the 20th EAHP-SH Symposium and Workshop took place and were (pre-)published more than a year after the event. In this workshop report as well as in the four related session reports, we have applied nomenclature of the revised 4^th^ edition of the WHO Classification as in place at the time of the workshop and refer to both updates (5^th^ edition WHO Classification for Haematolymphoid Tumours [WHO-HAEM5] and the International Consensus Classification [ICC]) where relevant (https://tumourclassification.iarc.who.int) [5, 6]. 

In this report, we address two pertinent differential diagnostic topics: the overlap with classic Hodgkin Lymphoma, including EBV-infected NLPHL, and the distinction or relationship between NLPHL and THRLBCL.

## Overlap with classic Hodgkin lymphoma

The majority of cases of NLPHL exhibit characteristic clinical, morphological, and immunophenotypical features of the entity. The disease can present at all ages but has a predilection for adolescents and young adults. Over 60% of the cases present as stage I or stage II disease, but in contrast to CHL less often restricted to supradiaphragmatic sites [7, 8]. Mediastinal and bone marrow involvement is less often seen. The growth pattern is classically nodular, supported by follicular dendritic meshworks and composed of small lymphocytes with a minor component of large neoplastic LP cells. However, the morphological and immunophenotypical spectrum of NLPHL is wide, resulting in difficulties distinguishing NLPHL from histopathologic mimics, including CHL and especially LRCHL. [9]

At the architectural level, NLPHL with predominantly nodular patterns A, C, and D share an overall nodular architecture with LRCHL, and moreover both tumor cells of NLPHL, pattern C, and LRCHL are both typically located in the periphery of nodules of mantle zone-type B-cell adding to similar morphological picture in HE overview [10]. At the immunophenotypical level, differences are larger. In contrast to CHL, the tumor cells of NLPHL have a complete B-cell phenotype with the expression of germinal center markers including BCL6, HGAL, and LMO2, but not CD10. CD30, CD15 and EBV are classically negative. More recently, MEF2B positivity and lack of expression of STAT6 and GATA3 have been shown to aid in the diagnostic distinction between NLPHL and CHL [11, 12]. Specifically, MEF2B, a member of the BCL6 transcriptional complex, is reported to be expressed in all cases of NLPHL, but negative in all cases of CHL. It should be noted that expression may be characteristic, but not specific since MEF2B is also expressed in most cases of follicular lymphoma, diffuse large B-cell lymphoma, and primary mediastinal B-cell lymphoma [13]. Other small B-cell lymphomas may more frequently be negative. While nuclear STAT6 (and its phosphorylated form pSTAT6) expression was reported in > 80% of CHL, no cases of NLPHL showed nuclear expression in the study by van Slambrouck et al. [12] Others report up to 37% expression of nuclear pSTAT6 in NLPHL, however. Expression was reportedly not related to variant patterns and clinical parameters of outcome [14]. Various antibodies to STAT6 and pSTAT6 are available with somewhat different specificity and sensitivity as well as (weak) cytoplasmic staining properties that should be considered when using these antibodies in diagnostic settings [12]. GATA3 expression may also assist in resolving this differential diagnosis, being positive in approximately 80% of CHL and generally negative in NLPHL [15]. Similar caveats on specificity and sensitivity apply, however.

The composition of the non-malignant infiltrate in NLPHL may add to differential diagnostic dilemmas between NLPHL and LRCHL and also to some extent to angioimmunoblastic T-cell lymphoma (AITL, WHO-HAEM5/ICC nodal T-follicle helper cell lymphoma, angioimmunoblastic type). All share the predominance of follicle helper T-(TFH) cells with a propensity to cluster around large B-cells [16]; in NLPHL and LRCHL, non-malignant TFH-cells surround tumor B-cells; in AITL, malignant TFH-cells surround EBV-positive or -negative non-malignant B-blasts [9, 17–19]. The phenomenon of “rimming” is therefore by no means specific for NLPHL, while being characteristic.

Therefore taken together, the prototypical immunophenotype of NLPHL and CHL differs in fundamental aspects (especially complete/incomplete B-cell phenotype), supporting the current thinking to fully separate the classification of these clinicopathological entities. In case of aberrant features, differential diagnostic considerations emerge.

Nineteen cases were submitted to the workshop that illustrated (among others) the overlapping features between NLPHL and CHL. The most common issue was an unusual immunophenotype (the absence of a complete B-cell phenotype, expression of CD30 or CD15, Supplementary Table S1).

**Fig. 1:**
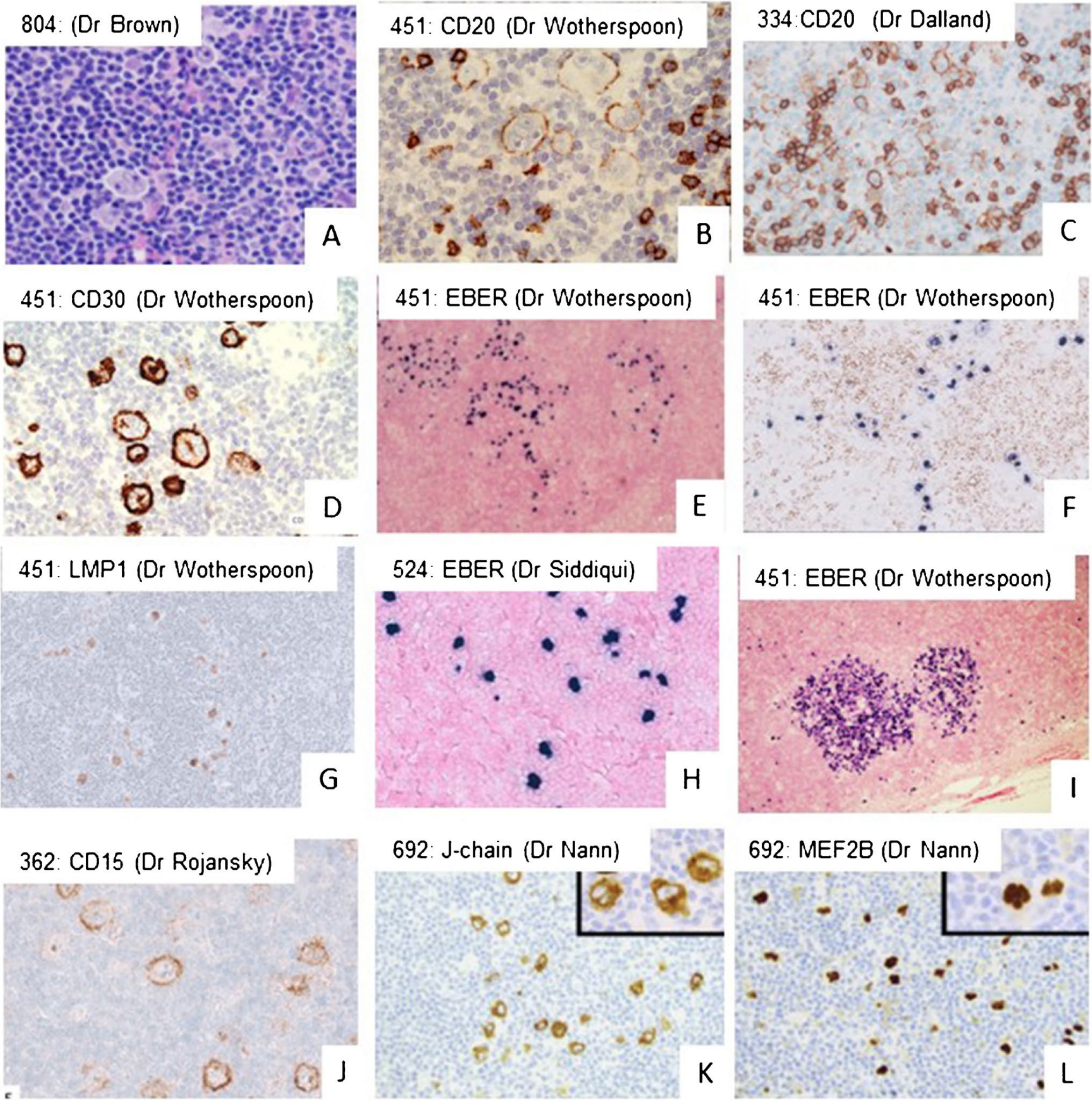
NLPHL cases with overlapping features to classic Hodgkin lymphoma. A Morphology of a case with overlapping features between NLPHL and CHL showing Fan patterns B and F (case 451, Dr A Wotherspoon, Royal Marsden Hospital, London, UK); B multinucleated LP tumor cells and with varying weak expression for CD20 may cause differential diagnostic issues to CHL and are often seen in EBV-positive cases (case 451); C also outside the context of EBV, CD20 may not always be strong and uniform (case 451); D strong CD30 expression may occasionally be seen in NLPHL both in EBV-positive and EBV-negative cases (case 451); E EBER expression in NLPHL in both large LP cells and small background lymphocytes (case 451); F in CD20/EBER double staining shows expression of EBER restricted to the large and small cell infiltrate (case 451); G EBV latency II with expression of LMP1 is the most frequent pattern in NLPHL (case 451); H in rare case, EBER expression is restricted to LP cells only (case 524, Dr I Siddiqi, University of Southern California, LA, USA); I EBV-positive follicular hyperplasia was observed in an unique case of NLPHL. An underlying immunodeficiency was excluded, and the finding is interpreted as a coincidental one (case 451); J CD15 expression in a case of NLPHL with Fan pattern A and C (case 362, Dr R Rojanski, Stanford University, Stanford, USA); K expression of J-chain (case 692, Dr D Nann, Tübingen, Germany) and L: MEF2B may help to distinguish NLPHL from CHL (case 692)

Twelve cases illustrated unusual expression of EBV. Eight of these were examples of EBER expression in the large cells (#524, #451, #798, #801, #567, #274, #692, #428). Detailed information is listed in Table 1. The majority presented as low stage disease and only 2 cases had stage III disease. No bone marrow involvement was noted. Therefore, clinical aspects were not remarkably deviant from prototypical NLPHL. While four cases showed pattern “A” morphology, patterns “C” and “D” were noted in four other cases. Immunophenotypical patterns are illustrated in Fig. 1 with examples from various submitters. As expected in the EBV context, CD30 was generally positive in all tumor cells (7/8). Remarkably, 3/5 cases showed a varyingly defective B-cell phenotype with decreased expression of CD79a and partial lack of BOB1, sometimes in combination with diminished or absent expression of OCT2 or PAX5. However, CD20 and OCT2 expression was usually strong (7/8). CD15 was negative in all instances. In addition to EBER positivity, the large cells expressed LMP1 (6/8) but were negative for EBNA2 (sporadic small cells in 1/6 cases tested, consistent with a EBV latency type II). These findings are largely in line with those reported in the literature indicating similar classic morphology, occasional lack of individual B-cell antigens, CD30-expression, and a more Reed-Sternberg-like morphology of the tumor cells in EBV-positive NLPHL [9, 20, 21]. Four additional cases that were submitted (#527, #675, #249, #338) as examples of NLPHL in which a significant infiltrate of small EBER-positive B-cells was noted. No expression in large tumor cells was seen and a latency 1 pattern was demonstrated. In the cases where EBV infects the large neoplastic cells, EBV may be playing a direct role in oncogenesis, while in these latter 4 cases, it may reflect a secondary or even bystander role.

**Table 1:** Cases of EBV-associated NLPHL submitted to the 20th EHP-SH workshop

Presenter	Imran Siddiqi	Andrew Wotherspoon	Flavia Ultimescu	Daniel Boyer	Irina Shupletsova	Candace Y. Reveles	Dominik Nann	Rohit Sharma	Sufang Tian	Cristiane R. Ferreira	Joshua Menke	Marc G.Evans
Affiliation	Southern Cal, USA	Royal Marsden, UK	Bucharest, Roumania	Michigan, USA	Moscow, Russia	Stanford, USA	Tubingen, GE	MSKCC, USA	Wuhan, China	SaoPaulo, Brasil	Stanford, USA	Harvard, USA
Age	75	61	11	59	40	70	38	37	11	50	67	19
Gender	M	M	M	M	M	F	F	M	M	M	M	M
Presentation	Stage II	Stage I	Stage I	Stage III, spleen	Stage II/III	Unknown	Stage I	Stage I/II	Stage II	Stage II, spleen	Stage IV, spleen	Stage II
Diagnosis	NLPHL, A + C	NLPHL, A	NLPHL, A	NLPHL, A	NLPHL, C + D	NLPHL,C	NLPHL, C + D	NLPHL, C (+ D)	NLPHL, A	NLPHL, D + E	NLPHL A + B (+ D)	NLPHL A + C
EBER	Large cells	Large cells	Large cells	Large cells	Large cells	Large cells	Large cells	Large cells	Small cells	Small cells	Small cells	Small cells
LMP1	Negative	Positive	Positive	Positive	Positive	Negative	Positive	Positive	nd	Negative	Negative	Sporadic small cells
EBNA2	Negative	Negative	Sporadic small cells	Negative	nd	Negative	Negative	nd	nd	Negative	Negative	Negative
B-cell phenoype	CD20, CD79a, PAX5, OCT2, BOB1 positive	CD20, CD79a, PAX5, OCT2, BOB1 positive	CD20, PAX5 positive CD79a, BOB1, OCT2 negative	CD20, OCT2 positive CD19, CD79a, PAX5, BOB1 weak/partial	CD20, CD79A, PAX5, OCT2 positive BOB1 weak	CD20, CD79am OCT2, BOB1 positive PAX5 weak	CD20, PAX5, BOB1, OCT2 positive CD79a weak	CD20, PAX5, CD19, CD22, OCT2, BOB1 positive	CD20, CD79a, PAX5, OCT2, BOB1 positive	CD20, CD79a, PAX5, OCT2, BOB1 positive	OCT-2, BOB.1 positive Pax5, CD79a weak, CD20 negative	CD20, CD79a, PAX5, OCT2, BOB1 postive
CD30	Positive	Positive	Positive	Positive	Positive	Positive	Positive	Negative	Negative	Negative	Negative	Positive
CD15	Negative	nd	Negative	Negative	Negative	Negative	Negative	Negative	nd	nd	Negative	Negative

*nd*, not done

In none of the 12 submitted cases, evidence of immune deficiency/dysregulation could be demonstrated when scrutinizing information on medical history and medication (within the limitations of cases submitted to a diagnostic workshop). The age range was wide (11–75 years) with half of the patients 50 years or older. In summary, the role of EBV in NLPHL including possible underlying immune dysregulation is unknown and remains a subject for future research.

During the panel review, the most helpful diagnostic feature in the delineation of NLPHL from LRCHL and EBV-positive lymphoproliferative disorders was found to be the growth patterns as indicated by Fan et al. [1], since these are unique to NLPHL, whereas the immunophenotype can sometimes be deviant with lack of mature B-cell markers. Also strong expression of OCT2 was considered a helpful feature. Among the other cases with rare immunophenotypes, there were four cases with CD15 expression in LP cells. All showed a complete B-cell immunophenotype without expression of CD30 and without EBV infection (4/4). Three out of 4 cases were IgD-negative. Two of these showed Fan pattern “D”, one case had combined patterns “A” and”C”, and one case combined patterns “D” and “E”. These cases were not considered to pose a significant differential diagnostic difficulty as their histological architecture, LP cellular morphological, and other immunophenotypical features were fully in line with NLPHL. Therefore, single aberrant features should not keep one from making a diagnosis of NLPHL, and multidisciplinary information and multiparameter histological information should always be utilized completely to come to a diagnosis of NLPHL.

## The impact of Fan patterns and the borderland of NLPHL and THRLBCL

In previous studies comparing NLPHL cases pattern E and THRLBCL, only minor differences between these two lymphoma types were observed when gene expression of the tumor cells, genomic aberrations or mutations were analyzed [18, 22–24]. Since detailed tumor cell molecular analyses are hampered by the low tumor cell content in the tissue, the above-mentioned studies involve limited case numbers. In contrast, major differences in the composition of the microenvironment were observed when NLPHL pattern A were compared with THRLBCL [25]. However, data comparing the microenvironment of NLPHL pattern E and THRLBCL are lacking so far.

The nature of clinical information available with the submitted cases did not allow for a comprehensive study on the impact of all individual Fan patterns on relapse risk and outcome, but did provide the opportunity for an exploratory analysis.

Firstly, cases classified as NLPHL were systematically assigned to Fan patterns based on morphological features and supported by CD20 and FDC (CD21 and/or CD23) immunohistochemistry to reliably assess the distribution of LP cells. Twenty-three cases showed a single pattern (pure A (*n* = 9), pure C (*n* = 4), pure D (*n* = 2), pure E (*n* = 8)) and combination of two patterns in 22 cases and 3 patterns in 5 cases. These numbers are largely in line with those reported, but should be interpreted with caution due to selection bias of cases submitted to a diagnostic workshop [25]. The observation of frequently combined Fan patterns emphasizes that core needle biopsies may preclude accurate pattern identification. Thus, basing clinical or prognostic conclusions on patterns in such limited samples should be done with great caution. Obviously, this does not take away from the major importance of recognizing Fan patterns to support a diagnosis of NLPHL.

All cases were reviewed with regard to the diagnostic criteria of THRLCBL; however, there was only one case (#269) fulfilling the criteria for a THRLBCL without any parts of accompanying NLPHL. Surprisingly, this patient had a history of a cHL 4 years prior to the diagnosis of THRLBCL.

Next, 21 cases with sufficient clinical information were submitted to illustrate the challenging differential diagnosis of NLPHL pattern “E” (THRLBCL-like) and THRLBCL and were included to explore this differential diagnosis and treatment implications. The cases were selected based on the predominant diffuse growth pattern and the T-cell and histiocyte-rich background infiltrate as well as the availability of clinical information. The panel then decided not to approach the cases with the pre-set diagnoses of NLPHL pattern E and THRLBCL but to divide them into two groups, those with good clinical outcome and those that were refractory to therapy. Clinical features are detailed in Supplementary Table 2. Cases were divided into two subgroups: (i) responders, cases with response to first line treatment and favorable clinical outcome, and (ii) non-responders, patients with primary refractory disease or early relapse. Progress or relapse within the first 24 months after initial diagnosis has been identified to be the most important adverse prognostic factor in NLPHL, as in other lymphomas, and therefore serves as a justifiable surrogate study end-point [26]. Eighteen of the 21 cases were treated by R-CHOP. The other three patients had received R-CVP (*n* = 1) or R-ICE (*n* = 1), and one 6-year old boy was followed by watch-and-wait. Nine of the R-CHOP treated patients were primary refractory, whereas the other 12, including the patients treated with R-ICE and the boy under watch-and wait, achieved complete remission. Between the refractory group and the responders, there were no significant differences in age (mean age 41 vs 34 years, refractory vs responders, ranges: 7–80 years versus 6–60 years, *p* > 0.05). The patients with refractory disease presented more frequently with advanced stage with four patients in stage IV and five patients in stage III compared with the responders seven patients in stages I–II, three patients in stage III, and two patients in stage IV). There was also a trend towards a higher rate of bone marrow involvement in 4 of 9 refractory patients (44%) versus 2 of 12 patients (16.6%) with bone marrow infiltration in the responders group. The small number of patients preclude meaningful statistical significance, however. Male gender was also slightly more frequent in the refractory group: The refractory patients were males only (9/9) compared with 8/12 (75%) males in the responders group.

**Fig. 2:**
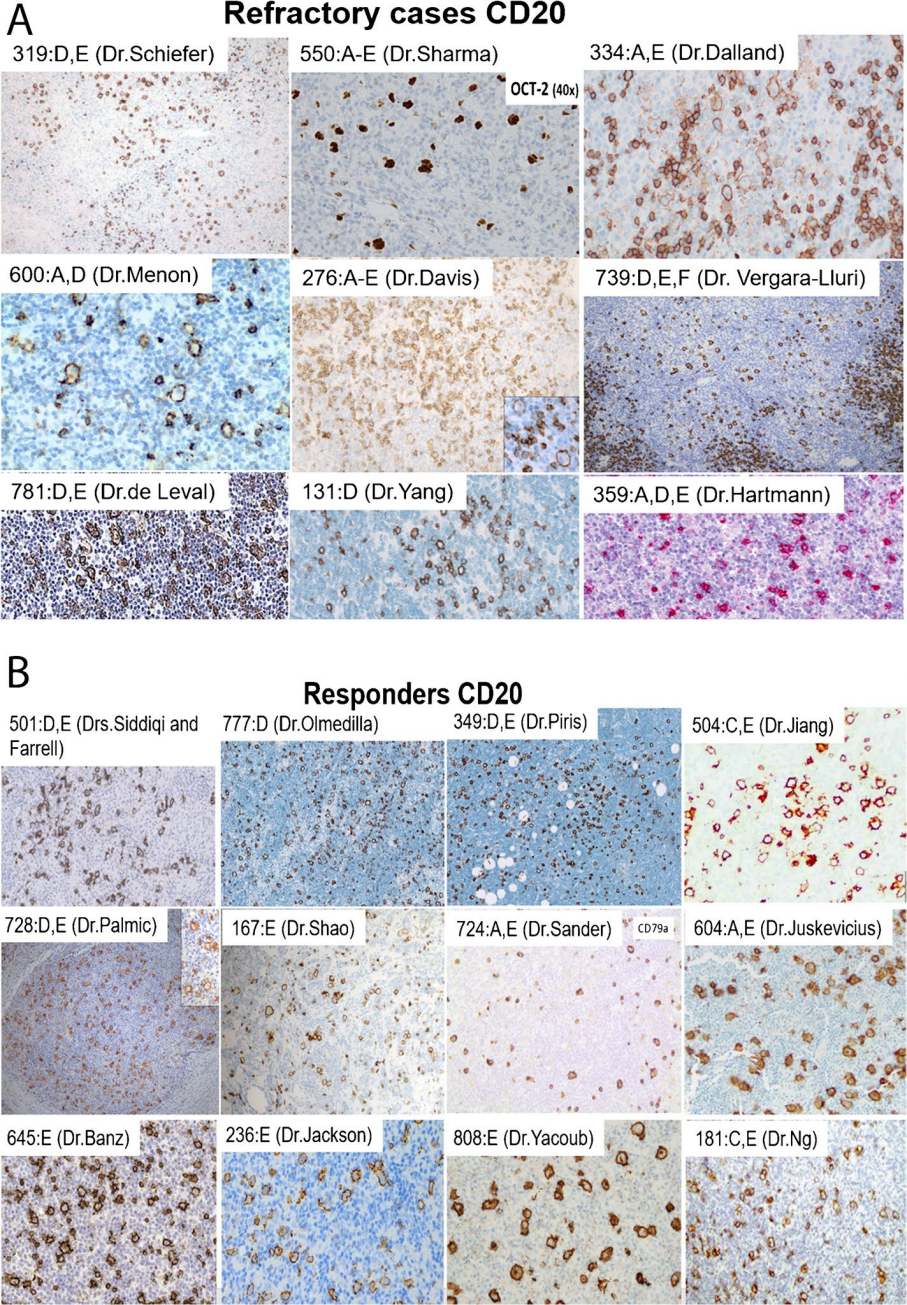
Examples of NLPHL cases with variant growth patterns (D/E) at the border to THRLBCL. A Examples of CD20-immunostainings (and OCT2-immunostaining in case 550, Dr R Sharma, Memorial Sloane Kettering Cancer Center, NY, USA) of refractory patients, highlighting single scattered LP cells with only little reactive B-cells in the background. B Examples of CD20-immunostainings of responding patients, highlighting single scattered LP cells with hardly any little reactive B-cells in the background. Case numbers, Fan patterns, and names of submitters are indicated with the images and in Table 2

Due to the small cohort size, no general conclusions on stage and gender can be drawn. However, in previous studies, bone marrow infiltration has been recognized to be relatively frequently associated with a fatal course of disease [27], which is otherwise unusual in NLPHL. The two session moderators (SH, DDJ) systematically reviewed all cases for Fan patterns, tumor cell phenotype, and composition and patterns of microenvironment T-cell and histiocytic populations. All features were scored independently, and discrepancies were resolved by consensus, fully realizing poor reproducibility of microenvironment population scoring using methods other than automated image analysis [28], which was beyond the scope of this EAHP-SH Workshop review. In most of the cases, PD1-positive T-cell rosettes surrounding the neoplastic LP cells were detected, even in cases with extensive Fan pattern “E” areas. Neither the tumor cell content nor the number of accompanying small reactive B-cells differed between the responders and refractory group by visual inspection. Assessment of infiltration patterns in this series of the refractory cases suggested that these frequently showed some remnants of small B-cells surrounding extensive areas of pattern E (Fig. 2). Unexpectedly, in the responders group, the amount of reactive B-cells seemed rather lower than in the refractory group; however, this was not statistically significant.

**Fig. 3:**
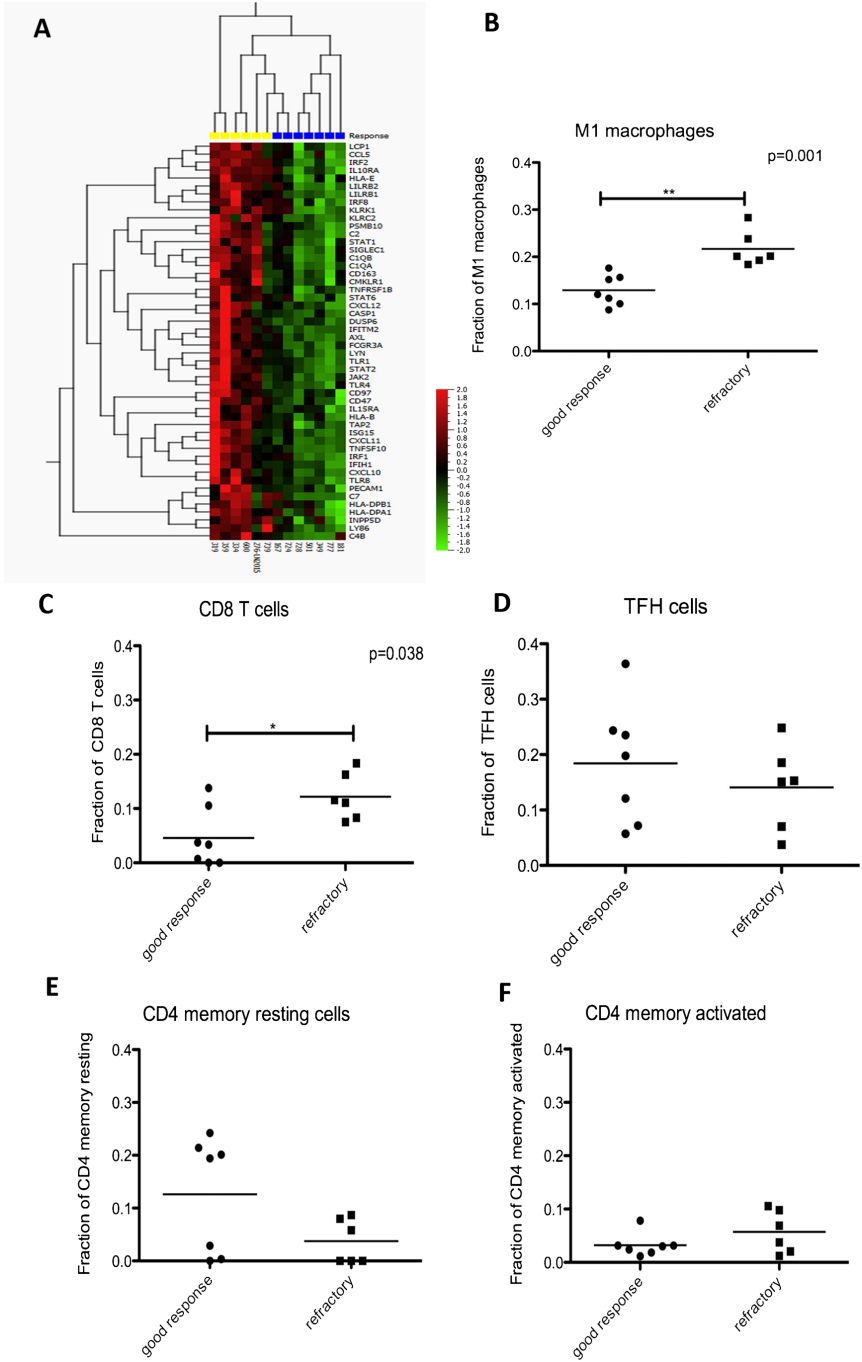
Characterization of thirteen cases of NLPHL variants at the border to THRLBCL by gene expression profiling.** A** Supervised clustering of six refractory NLPHL cases (yellow) and seven responding cases (blue) according to the 50 most differentially expressed genes in the Nanostring PanCancer Immune Panel. **B** Cibersort of the Nanostring PanCancer Immune Panel data revealed a significantly higher number of M1 macrophages in the refractory patients (*p* = 0.001, Mann–Whitney test).** C** Cibersort of the Nanostring PanCancer Immune Panel data revealed a significantly higher number of CD8^+^ T-cells in the refractory patients (*p* = 0.038, Mann–Whitney test).** D** Cibersort of the Nanostring PanCancer Immune Panel data revealed no significant difference in the number of T_FH_ cells.** E** Cybersort of the Nanostring PanCancer Immune Panel data revealed no significant difference in the number of CD4^+^ memory resting cells.** F** Cibersort of the Nanostring PanCancer Immune Panel data revealed no significant difference in the number of CD4^+^ memory activated T-cells

We further aimed to characterize differences between the refractory and responders groups by applying an exploratory molecular immune cell profiling assay (JR, WK). The Nanostring Pancancer Immunepanel (Nanostring, Seattle, WA, USA) was applied to 13 cases, comprising six refractory patients and seven responders, yielding gene expression values for 770 immune-related transcripts in the microenvironment. Clinicopathological details are listed in Table 2. One hundred and thirty differentially expressed genes (*p* < 0.05) between the refractory and responders groups were identified. In the refractory group, these contained genes related to an inflammatory immune response with a high number of innate immune response genes, NK-cells, IFN-gamma signalling, complement factors, M1 macrophages, and CD8 cells, indicating a prominent host response in the refractory patients (Fig. 3). We applied a CIBERSORTx analysis [29] to deconvolute the expression data into immune cell populations. Type M1 macrophages as well as CD8 + T-cells were found to more abundant in the microenvironment of the biopsy material of refractory patients when compared with responders with a mean 22% M1 type macrophages in the refractory group compared with mean 13% in the responders group (*p* = 0.0012, Mann–Whitney test). Likewise, there was a mean of 12% CD8 + T-cells in the refractory group compared with 5% in responders group (*p* = 0.0381, Mann–Whitney test, Fig. 3). These statistically significant but rather subtle quantitative differences of specific cell populations cannot easily be perceived by visual inspection and will, thus, not be directly translatable into daily practice. While these findings may be a starting point for further research, biological interpretation may not be straight forward. These data reflect previous findings of high numbers of CD8-positive cells and macrophages in the microenvironment of THRLBCL [30, 31]. However, the spectrum of CD4:CD8 ratios in the microenvironment of THRLBCL is likely quite wide [18, 30]. At a more detailed level, CD8-positive T-cells in THRLBCL have a close interaction with the tumor B-cells, and this probably represents a cytotoxic antitumor immune response [31]. However, the interactions may be significantly more complex with a role for high numbers of PDL1-positive macrophages which are also seen in close vicinity to the tumor cells. [30]

**Table 2:** Cases of NLPHL pattern E and THRLBCL included in the exploratory molecular study on immune microenvironment markers predictive for treatment outcome

Good response							
Case	501	724	777	181	167	349	728
Submitter	Daniel Farrell	Birgitta Sander	Gabriel Olmedilla	Siok Bian Ng	Haipeng Shao	Miguel A. Piris	Patricia M. Palmic
	Los Angeles, US	Stockholm, Sweden	Madrid, Spain	Singapore	LeeMoffit, US	Madrid, Spain	Paris, France
Age	48	37	8	6	55	60	25
Gender	F	M	M	M	M	F	M
Diagnosis	NLPHL, D, E	NLPHL, A, E	NLPHL, D, E	NLPHL, C, E	NLPHL, E (with fibrosis)	NLPHL, E	NLPHL, D, E
Complete B-cell phenotype	+	+	+	+	+	+	+
CD30	Not done	-	-	Partly	Not done	-	-
CD15	Not done	-	-	-	Not done	-	Not done
EBER	Not done	-	-	-	-	-	-
IgD	Not done	Not done	Not done	+	-	Not done	+
Clinical presentation	Perihepatic lymphadenopathy for 7 years, slowly progressive to lymphadenopathy above and below mediastinum	Left axillary ln and 2 hypermetabolic foci in the spleen	Right inguinal Lymphadenopathy	Multiple lymph nodes and spleen	Enlarged cervical lymph node for several years then weight loss, liver failure, cytopenias	Generalized lymphadenopathies	Neck nodes slowly growing for5 months, nodes both sides of diaphragm
Stage	II	II	I	III	II	IV	III
Splenomegaly	+	+	-	+	-	-	+
Auto-immune disease	-	-	-	-	-	RA	Crohn
First-line treatment	6 × R-CHOP	R-CHOP	Watch and wait	4 × RICE	6 × R-CHOP	6 × R-CHOP	6 × R-CHOP
Treatment outcome and follow-up	Complete remission (4 years)	Complete remission (2 years)	Watch and wait, stable disease (3 years)	Complete remission	Complete remission	Complete remission	Complete remission
Poor response							
Case	276	319	334	359	739	600	
Submitter	Adam R. Davis	Ana-Iris Schiefer	Joanna C. Dalland	Sylvia Hartmann	Maria Vergara-Lluri	Madhu Menon	
	Philadelphia, US	Vienna, Austria	Rochester, US	Frankfurt, Germany	Los Angeles, US	Detroit, US	
Age	33	27	57	46	30	7	
Gender	M	M	M	M	M	M	
Diagnosis	NLPHL, E	NLPHL, C, D, E	NLPHL, A, E/THRLBCL	NLPHL, A,D,E/THRLBCL	NLPHL, D, F, E	NLPHL, A, D, E	
Complete B-cell phenotype	+	+	+	+	+	+	
CD30	Weak	-	Weak	-	-	-	
CD15	-	+	-	+	-	-	
EBER	-	-	-	Not done	-	-	
IgD	Not done	-	-	-	Not done	-	
Clinical presentation	Stage IV	Generalized lymphadenopathy, hepatosplenomegaly and axial skeleton involvement, B-symptoms, bone marrow involvement	Bilateral tonsils, right axilla, spleen, and bone lesions involving the cervical, thoracic, lumbar spine, and pelvis	Multiple lymph nodes and spleen	Multiple lymph nodes, B-symptoms	Diffuse lymphadenopathy and numerous hypermetabolic liver lesions	
Stage	IV	IV	IV	III	III	III	
Splenomegaly	+	+	+	+	-	-	
Auto-immune disease	CVID	-	-	-	-	-	
First-line treatment	R-CHOP	8 × R-CHOP	R-CHOP	2 × R-CHOP	6 × R-CHOP	R-CHOP	
Treatment outcome and follow-up	Relapse (spleen)	Refractory disease	Refractory and progression of bone lesions	Refractory. 3 × R-DHAP, 1 × R-ICE, autologous SCT	Relapse after 4 months, ICE, allogeneic SCT	Refractory, DLBCL diagnosed after 9 months

Taken together, it can be concluded that with the currently available techniques, objective differentiation between the Fan pattern E of NLPHL and THRLBCL as a means to predict aggressive behavior cannot yet be reliably made. However, this pilot study suggests that biological differences are, at the basis of the clinical behavior, ready to be identified in large, well-controlled, multicenter studies. Alternatively, modern technology may reveal newer insights that may better correlate to clinical outcome than the currently applied Fan patterns. For current daily practice, the EAHP/SH Workshop panel recommends to name Fan patterns in order to collect more data and to learn about the nature of these different patterns in a large multicenter cohort. Collection of global-wide cohorts like by the Global NLPHL One Working Group (GLOW; https://glowconsortium.org/) may answer questions on the clinical variability of different individual patterns.

## Summary

In the Session 4 of the 20th meeting of the European Association for Haematopathology, we concluded that growth patterns in NLPHL have an important diagnostic value. NLPHL cases with aberrant phenotypes of the LP cells do exist (CD30-positive, CD15-positive, or EBV-positive) and may be challenging to differentiate from LRCHL or EBV-associated lymphoproliferations. In such cases, recognition of Fan patterns is a very helpful diagnostic feature. We, furthermore, studied variant pattern NLPHL cases with a morphology at the border between NLPHL and THRLBCL, frequently presenting at an advanced stage. In the studied cohort, the morphologic features were not helpful to predict therapy response in R-CHOP treated patients. In contrast, the immune microenvironment differed by gene expression between chemo-immunotherapy responding and refractory patients. However, this observation cannot yet be readily transferred to immunohistochemically support pathological observations in routine practice.

## Take home messages

- Recognition of Fan patterns is very helpful to support a diagnosis of NLPHL and differentiate NLPHL from its differential diagnoses.
- Expression of a complete B-cell immunophenotype and especially strong OCT2 expression supports a distinction of NLPHL from CHL.
- Aberrant expression of single immunohistochemical makers in otherwise prototypical NLPHL is not uncommon and should not preclude a diagnosis of NLPHL.
- Different Fan patterns, especially variant Fan patterns (C, D, E, F), are frequently found combined within a single case. As a group, variant Fan patterns have been reported to be associated with shorter time-to-relapse compared to typical patterns (A, B). Currently insufficient data on individual variant patterns are available, precluding clinical decisions based on Fan patterns alone.
- NLPHL with Fan pattern E and THRLBCL are part of the same spectrum, and the distinction is poorly reproducible. As currently defined, a diagnosis of NLPHL with Fan pattern E versus THRLBCL is insufficiently predictive of clinical aggressive behavior by itself. Decisions on clinical management supported by discussion in a multidisciplinary tumor board are therefore strongly recommended.
- The cellular composition of the tumor microenvironment may impact on clinical outcome in NLPHL with Fan pattern E and THRLBCL, irrespective of pathological classification, which should be further investigated in larger patient cohorts

**Table:** 

**Profiling immune cells to differentiate between good-response and poor-response patients with NLPHL-THRLBCL-like and THRLBCL morphology**
RNA was isolated from formalin-fixed paraffin-embedded unstained slides as submitted to the EAHP-SH Workshop according to standard methods using the RNeasy FFPE kit (Qiagen, Hilden, Germany) following the manufacturer’s instructions. Digital-multiplexed gene expression (DMGE) profiling was performed with Nanostring Pancancer Immune Profiling Panel (NanoString Technologies, Seattle, WA, USA) platform, a 770-plex gene expression panel to measure the human immune response.20 In brief, 200 ng purified RNA was used as input material. The custom codeset was hybridized to the total RNA overnight, before being purified and fixated using the Nanostring Prep Station. Gene expression data were obtained using the Immunepanel nCounter Digital Analyzer, which automatically performs quality control, normalization, and data analysis. Data quality was assessed by the ratio of preset fields of view (FOV) to observed FOV, which should not be below 0.8. The FOV were set to a high resolution of 555. In addition, only samples with a binding density between 0.1 and 2.25 were included. Raw data QC and normalization were done using nSolver software (NanoString Technologies, version 4.0). For differential gene expression analyses using two group analyses Qlucore software (Qlucore, Lund, Sweden; version 3.6) was applied. Next, deconvolution was performed using Cibersort to assign gene expression profiles to immune cell populations (http://cibersort.stanford.edu/).21

### Supplementary information

Below is the link to the electronic supplementary material.

Supplementary file1 (XLSX 17 KB)

Supplementary file2 (DOCX 29 KB)

## Data Availability

Gene expression raw data can be made available upon request by contacting the first and last authors of this paper (SH, DDJ).
