# Supplementary material for: The spectrum of nodular lymphocyte predominant Hodgkin lymphoma: a report of the lymphoma workshop of the 20th meeting of the European Association for Haematopathology
Source: Virchows Arch. 2023 Aug 2;483(4):451–63. doi: 10.1007/s00428-023-03554-1 (PMC10611620; doi:10.1007/s00428-023-03554-1)
Supplement: Supplementary file 2 — Supplementary file2 (DOCX 29 KB) [file 428_2023_3554_MOESM2_ESM.docx]

**Table S1: All cases of NLPHL pattern E and THRLBCL for which sufficient clinical information was submitted to allow clinico-pathological correlations**
